# Resistant Starch Alters the Microbiota-Gut Brain Axis: Implications for Dietary Modulation of Behavior

**DOI:** 10.1371/journal.pone.0146406

**Published:** 2016-01-08

**Authors:** Mark Lyte, Ashley Chapel, Joshua M. Lyte, Yongfeng Ai, Alexandra Proctor, Jay-Lin Jane, Gregory J. Phillips

**Affiliations:** 1 Department of Immunotherapeutics and Biotechnology, School of Pharmacy, Texas Tech University Health Sciences Center, Abilene, Texas, 79606, United States of America; 2 School of Medicine, Texas Tech University Health Sciences Center, El Paso, Texas, 79905, United States of America; 3 Department of Food Science and Human Nutrition, College of Human Sciences, Iowa State University, Ames, Iowa, 50011, United States of America; 4 Department of Veterinary Microbiology & Preventive Medicine, College of Veterinary Medicine, Iowa State University, Ames, Iowa, 50011, United States of America; Western University of Health Sciences, UNITED STATES

## Abstract

The increasing recognition that the gut microbiota plays a central role in behavior and cognition suggests that the manipulation of microbial taxa through diet may provide a means by which behavior may be altered in a reproducible and consistent manner in order to achieve a beneficial outcome for the host. Resistant starch continues to receive attention as a dietary intervention that can benefit the host through mechanisms that include altering the intestinal microbiota. Given the interest in dietary approaches to improve health, the aim of this study was to investigate whether the use of dietary resistant starch in mice to alter the gut microbiota also results in a change in behavior. Forty-eight 6 week-old male Swiss-Webster mice were randomly assigned to 3 treatment groups (n = 16 per group) and fed either a normal corn starch diet (NCS) or diets rich in resistant starches HA7 diet (HA7) or octenyl-succinate HA7 diet (OS-HA7) for 6 week and monitored for weight, behavior and fecal microbiota composition. Animals fed an HA7 diet displayed comparable weight gain over the feeding period to that recorded for NCS-fed animals while OS-HA7 displayed a lower weight gain as compared to either NCS or HA7 animals (ANOVA *p* = 0.0001; NCS:HA7 *p* = 0.244; HA7:OS-HA7 *p*<0.0001; NCS:OS-HA7 *p*<0.0001). Analysis of fecal microbiota using 16s rRNA gene taxonomic profiling revealed that each diet corresponded with a unique gut microbiota. The distribution of taxonomic classes was dynamic over the 6 week feeding period for each of the diets. At the end of the feeding periods, the distribution of taxa included statistically significant increases in members of the phylum *Proteobacteria* in OS-HA7 fed mice, while the *Verrucomicrobia* increased in HA7 fed mice over that of mice fed OS-HA7. At the class level, members of the class *Bacilli* decreased in the OS-HA7 fed group, and *Actinobacteria*, which includes the genus *Bifidobacteria*, was enriched in the HA7 fed group compared to the control diet. Behavioral analysis revealed that animals demonstrated profound anxiety-like behavior as observed by performance on the elevated-plus maze with time spent by the mice in the open arm (ANOVA *p* = 0.000; NCS:HA7 *p* = 0.004; NCS:OS-HA7 *p* = 1.000; HA7:OS-HA7 *p* = 0.0001) as well as entries in the open arm (ANOVA *p* = 0.039; NCS:HA7 *p* = 0.041; HA7:OS-HA7 *p* = 0.221; NCS:OS-HA7 *p* = 1.000). Open-field behavior, a measure of general locomotion and exploration, revealed statistically significant differences between groups in locomotion as a measure of transitions across quadrant boundaries. Additionally, the open-field assay revealed decreased exploration as well as decreased rearing in HA7 and OS-HA7 fed mice demonstrating a consistent pattern of increased anxiety-like behavior among these groups. Critically, behavior was not correlated with weight. These results indicate that diets based on resistant starch can be utilized to produce quantifiable changes in the gut microbiota and should be useful to “dial-in” a specific microbiome that is unique to a particular starch composition. However, undesirable effects can also be associated with resistant starch, including lack of weight gain and increased anxiety-like behaviors. These observations warrant careful consideration when developing diets rich in resistant starch in humans and animal models.

## Introduction

The role of the microbiota in gut-to-brain communication is increasingly being recognized as a major determinant of behavior. Research both in humans and animal models has demonstrated that specific bacterial taxa are associated with and can induce alterations in cognitive states including anxiety and anxiety-like behavior [1–4]. For example, oral introduction of a novel bacterial species, *Campylobacter jejuni*, into mice resulted in the development of anxiety-like behavior in the absence of any detectable immune response [5, 6]. This bacterial-induced behavioral response was further shown to activate specific regions in the brain associated with anxiety-like behavior and the vagal afferents from the gut were involved [7]. Other studies have likewise shown that the microbiome can affect brain development and neurobehavioral issues [8, 9].

The majority of studies to date that have addressed behavior through modulation of the microbiota, also referred to as the microbiota-gut-brain axis, have principally relied on the administration of specific bacteria (i.e., probiotics) or through the complete transfer of the microbiome (i.e., fecal microbial transplantation) [9]. A more universal and hence more easily implemented approach to the modulation of the microbiota may be through the use of diet. A number of studies have demonstrated that dietary modulation can induce alterations in the composition of the microbiota (for review see [10]).

That dietary manipulations can affect neurological function was recently reported by Borre *et al*. [11] who showed that targeted dietary changes involving a broad range of chemical constituents resulted in neuroprotection and cognitive enhancement in a rodent model of neurodegeneration and depression. As such, development of targeted diets to influence brain function holds promise as a therapeutic modality that may reduce or replace the use of current pharmaceutical agents. The mechanisms by which such diets may function in influencing neurological function are still incompletely understood. Despite its potential significance, few studies have utilized diet as a means influence brain function and the microbiota-gut-brain axis by altering the microbiota. In the first study of on this topic, Li *et al*. [12] reported that mice fed a meat-supplemented diet displayed increased microbial diversity that was positively correlated with increased learning and recall memory in the meat-fed animals [12].

There continues to be interest in the health benefits of low-digestible carbohydrates have been termed “resistant starch” (RS) [13]. RS contains modified starch components that are neither digested nor absorbed in the small intestine but instead are fermented by the resident colonic microbiota to produce a variety of metabolites that can benefit the host [14, 15]. Diets rich in RS have been associated with improved health [16, 17] and RS diets are also known to alter the taxonomic composition of the gut microbiota in both animals [18–22] and humans [23–25].

Given the clear indication that gut bacteria can affect behavior and brain activity and the current interest in RS as a means to improve health, we sought to determine if diets rich in RS induced alterations in the composition of the microbiota that were also associated with changes in behavior. For this, we utilized a combination of culture-independent taxonomic profiling of the gut microbiota coupled with robust behavioral analyses of mice over a 6-week feeding period. The results of this study have implications for development of dietary regimens of RS in both human and animal models.

## Materials and Methods

### Animal use

All experiments were approved by Texas Tech University Health Science Institutional Animal Care and Use Committee prior to initiation of the work. Four-week old male Swiss-Webster mice were obtained from Charles River Laboratories (Wilmington, DE). Upon arrival to the testing facility, mice were randomized, ear tagged and housed in individually ventilated cages (Tecniplast, Buguggiate, Italy) at a density of 4 per cage. The cages were placed on a rack in two horizontal rows aligned next to each other to minimize variations in light and human traffic. All mice had access to food and water throughout the experiment ad libitum. Mice were housed in standard laboratory housing, including a plastic box, food, water bottles, and remained on a 12 hour light/12 hour dark cycle (LabDiet^®^ 5P14, St. Louis, MO, USA). All behavioral testing was done during the light phase (0800–1800 h) with a 24–48 hour rest period between behavioral testing for each group. During the first week, no behavioral testing was performed in order for the mice to habituate to their new surroundings. After the first week, the mice were randomly divided into three groups of 16 (4 cages per group) with each group being fed a specific diet (see sections below for detailed analysis of food nutritional content). Within each cage, the diet was contained in a 4 oz feeding jar (5.1 cm height, 7.6 cm diameter) with a stainless steel snap on cap with a 2.5 cm hole to allow access to the food. Mice were fed at 0800 daily and monitored for their health except if they were being used in behavioral testing that day, then they were fed at 1800 after the testing period to ensure there was no impact on the behavioral tests. Mice were weighed every other day at 0800 unless they were participating in a behavioral test that day, in which they were weighed after the testing was completed for the day.

### Diets

Normal corn starch (NCS, Cargill Gel ^™^) and high-amylose corn starch (HA7, AmyloGel^™^) were purchased from Cargill Inc. (Minneapolis, MN). 2-octenyl-1-succinic anhydride and other chemicals were purchased from Sigma-Aldrich Co. (St. Louis, MO) and used without further treatments. Since the objective of this study was to compare the effect of starch resistance in altering both microbiota and behavior we selected the NCS control diet over a standard mouse chow diet. Moreover, a chow diet may contain numerous ingredients not present in the HA7 or octenyl succinic high-amylose (OS-HA7) diets that would have potentially introduced confounding factors in attributing changes in microbiota or behavior to the resistance of the starches. To prepare OS-HA7, HA7 was modified using 10% (w/w, dry starch basis) OSA following the method of Zhang *et al*. [26]. The starch was suspended in distilled water (35%, w/w, dry starch basis) with the pH adjusted to 8.0 using a sodium hydroxide solution (3%, w/w) and the temperature at 35°C. OSA was added to the starch suspension by drops while the pH maintained at 8.0 and the temperature at 35°C. After the reaction completed, as indicated by stabilization of pH, the pH was adjusted to 6.5 using hydrochloric acid (1.0 M). The starch was separated by centrifugation, washed with distilled water (2X) and with 100% ethanol (2X), dried at 37°C, and then ground. The degree of substitution of the OS-HA7 was approximately 0.058 as previously reported Ai *et al*. [27]. After boiling with water (3X), the cooked NCS, HA7 and OS-HA7 starches were used to prepare the diets for the mice. The composition of the diets is shown in Table 1.

**Table 1:** Diet compositions.

Ingredient	Weight percentage (%)		
	NCS diet	HA7 diet	OS-HA7 diet
NCS ^a^^,^ ^b^	55.0	-	-
HA7	-	55.0	-
OS-HA7	-	-	55.0
Casein ^c^	20.0	20.0	20.0
Glucose	15.0	15.0	15.0
Mineral mix (AIN-93)	3.5	3.5	3.5
Choline	0.2	0.2	0.2
Methionine	0.3	0.3	0.3
Vitamin mix (AIN-93)	1.0	1.0	1.0
Corn oil	5.0	5.0	5.0
Total	100.0	100.0	100.0

^a^ The ingredients were all weighed on as-is weight basis.

^b^ NCS: normal corn starch, HA7: high-amylose corn starch, OS-HA7: HA7 modified with 10% (w/w, dry starch basis) OSA.

^c^ The non-starch ingredients were purchased from Harlan Laboratories (Madison, WI).

### Behavioral Testing

Behavioral testing consisted of testing on an elevated plus maze (EPM) and an open field apparatus. Animals were tested on the days immediately prior to sacrifice with each test being performed on a separate day and EPM performed as the first behavioral test. Specifically, two days before sacrifice, mice were tested a single time on the EPM. 24 hours after completion of the EPM, mice were subjected a single time to the open field behavioral assay. The following day, mice were sacrificed. The EPM has been extensively employed to measure anxiety-related behaviors in rodents [28]. The open field apparatus is a common measure of general activity and exploration [29]. All mice to be tested for the day were transported into the testing room 1 hour prior to testing to allow for habituation to the testing room. Mice were covered when transported from the housing room to the testing room to avoid any external stimuli. The testing room consisted of a 9 ft x 9 ft dimly lit room using five 15W light bulbs on a table in the corner of the room (30 lux). Animal tracking was done by the automatic tracking system AnyMaze (Version 4.98, Stoelting, MA). The autofocus high-definition video camera (Logitech C615, Newark, CA) was attached to a stand and positioned directly above the testing field out of view of the mouse. In order to eliminate variability in manually scored observations, all behavioral testing was conducted by the same observer. Testing on one apparatus was separated by at least 2 days before testing in a different procedure.

### Elevated Plus Maze (EPM)

The EPM consisted of a plus shaped apparatus with two arms closed by black Plexiglas walls and two arms left open to test for anxiety-like behavior in mice. The open and closed arms were 35.5cm in length and were elevated 62.5cm above the ground. The test mouse was placed in the center of the EPM facing a closed arm and to ensure that all mice were subjected to the same testing conditions, the same closed arm was used each time. The observer then moved to the corner of the room, out of sight of the test mouse, and manually scored the number of times the mouse groomed. The tracking program recorded the number of entries and time spent in the closed and open arms, which were used to determine changes in anxiety-like behavior among the groups. An entry into the arm was defined as the time when 80% of the mouse’s body was in the arm, which is equivalent to four paws in the arm. Behavioral testing lasted for 10 minutes. Fecal samples were collected at the end of each test by transferring the sample from the testing field into a sterile Eppendorf tube using sterile forceps. The forceps were cleaned with 70% ethanol after every use. The fecal samples were kept in a frozen Eppendorf tube holder in an ice chest until all experiments were finished for the day. They were then transported to a -84°C freezer. The EPM was cleaned with 70% ethanol between each test.

### Open Field Test

The test mouse was placed in the center of the open-field apparatus. The open-field apparatus consisted of a transparent Plexiglas (40.7cm x 40.7cm) box on top of a plastic base that was covered by a solid black plastic sheet. The open field apparatus was virtually divided, using the tracking system, into four equal segments, titled Zone 1 (upper left quadrant), Zone 2 (upper right quadrant), Zone 3 (lower left quadrant), and Zone 4 (lower right quadrant). Behavioral testing was performed for 5 minutes. During this time, the observer, located in the corner of the room, manually scored the number of times the test mouse reared, groomed and defecated. Rearing was defined as the two front paws off the ground while the two hind paws remain on the ground. The tracking program recorded the entries and time spent in each zone as well as the total distance travelled to determine if there were any differences in the locomotor activity levels. The apparatus was cleaned with 70% ethanol between every test and all fecal specimens were removed.

### Biological sample collection and analysis

#### Fecal specimens

Immediately before sacrifice, mice were individually placed into an empty, clean cage and allowed to voluntarily discharge fecal pellets that were promptly collected, stored in an Eppendorf tube, and then frozen in a -20°C frozen block. All samples were transferred into a -84°C freezer for long-term storage until analysis. Although all mice were noted to have produced fecal pellets, only fecal samples from 10 mice demonstrating the most consistent behavioral changes were chosen for microbiota analysis.

#### Hair Samples

Mice were anesthetized using isoflurane and remained under anesthesia via a face mask throughout the procedure. Using an electric razor, the hair was shaved off the sides, backs and stomachs of the mice and placed into a vial. Hormonal analysis using hair samples was performed as previously described using a high-pressure liquid chromatography-triple quadrupole mass spectrometry technique which allowed for the simultaneous measurement of all hormones [30].

#### Genomic DNA Isolation

Prior to genomic DNA isolation, fecal and cecal samples were stored at -80°C. Genomic DNA isolation was performed using the PowerSoil DNA Isolation Kit (MoBio, Carlsbad, CA) on samples from ten mice in each group for each time point as well as cecal contents. The manufacturer’s protocol was followed with the exception that the initial vortex step was extended to 20 minutes to thoroughly homogenize the samples. The purified genomic DNA extracts were quantified using a Qubit 2.0 Fluorometer (Life Technologies, Carlsbad, CA), and stored at -20°C in 10mM Tris buffer.

#### DNA Sequencing and Analysis

PCR amplification of the V4 variable region of the 16S rRNA gene using V4 region specific primers (515F-816R) and amplicon sequencing were performed by Institute for Genomics & Systems Biology at the Argonne National Laboratory (Argonne, IL) on the Illumina MiSeq Platform. The sequences were analyzed using QIIME (Quantitative Insights into Microbial Ecology) [31]. Briefly, reads were first demultiplexed and quality filtered, then sequences with homopolymer runs or ambiguous bases greater than 6, nonmatching barcodes, barcode errors, or quality scores less than 25 were removed. Operational taxonomic units (OTUs) were called using uclust and the closed reference OTU picking strategy in QIIME [32]. Sequences were aligned to the Greengenes (13_8) database using PyNAST at 97% similarity [33, 34]. Taxonomic assignments were made based on the Greengenes reference database using the RDP classifier 2.2 in QIIME [35, 36]. Phylogenetic trees, alpha diversity, beta diversity, principal coordinates plots (PCoA), Analysis of similarity (ANOSIM), and Adonis tests were also generated using QIIME. Kruskal-Wallis tests were coupled with Wilcoxon Rank Sum tests and performed on taxonomic summaries, obtained from the QIIME pipeline, using a custom R script (R Project) provided by the Institute for Genome Sciences at the University of Maryland School of Medicine [37].

### Data analysis of non-microbiome data

Initial statistical analysis on behavioral testing, hormone levels and weight loss data was performed using an ANOVA test to determine any significant difference among the three diet groups. Following an ANOVA test with a *p*-value <0.05, the Bonferroni test was performed to determine significant differences between NCS and HA7, NCS and OS-HA7 and HA7 and OS-HA7. Statistical significance was set at *p* <0.05.

## Results

### Differential Weight Gain among Resistant Starch Groups

**Fig 1:**
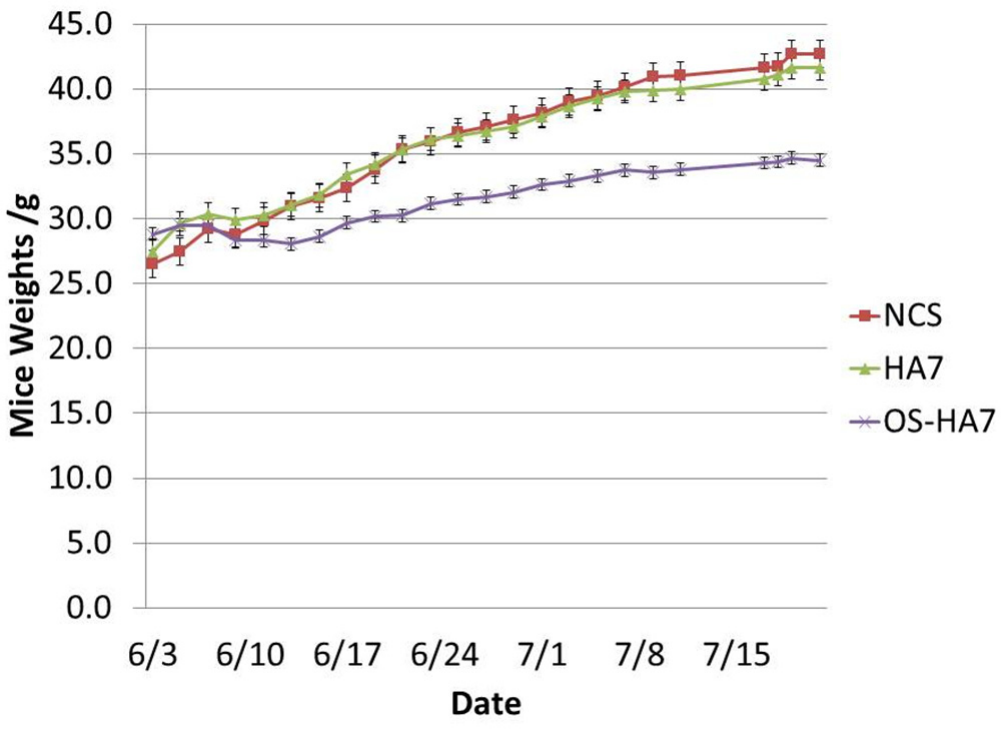
Average mice weight gain for the three diets over the duration of behavioral testing. The weights of the mice were determined every other day for the duration of testing. Values represent the mean weight in each group ± SEM, n = 16/group.

It was determined that there were no statistically significant differences in weight gain between the mice treated with NCS and HA7 with a lower weight gain for mice treated with OS-HA7 (ANOVA *p*<0.0001; NCS:HA7 *p* = 0.244; HA7:OS-HA7 *p*<0.0001; NCS:OS-HA7 *p*<0.0001) (Fig 1). The effect of differential rate of weight gain between the NCS or HA7 versus OS-HA7 did not elicit concern as the OS-HA7 was specifically designed to be the most resistant to digestion, making it inherently less bioavailable for absorption by the animal’s small intestine. Additionally, as the most dramatic behavioral response was found in the HA7 treatment group, it is important to note that the HA7 treatment group did not have a statistically significant difference in weight gained or trend of weight gain over time than did the NCS-treatment group. Moreover, although the OS-HA7 treatment group did have a different weight gain than the NCS treatment group, in behavioral analysis, there was not found to be a statistically significant difference between the NCS and OS-HA7 treatment groups.

### Unique Microbial Community Structure in RS-fed Animals

A total of 6,140,396 amplicon reads passed quality filtering and were submitted to closed reference OTU picking at a 97% similarity to the Greengenes database. Reads that failed to match an OTU in Greengenes were removed. 5,204,391 reads corresponded to 3,956 OTUs, with a mean of 43,734±34,742 reads per sample post OTU picking. Of 120 total samples, one sample from the HA7 fed group from week 6 failed to amplify during library preparation.

### Dietary induced changes in the microbiota over time

**Fig 2:**
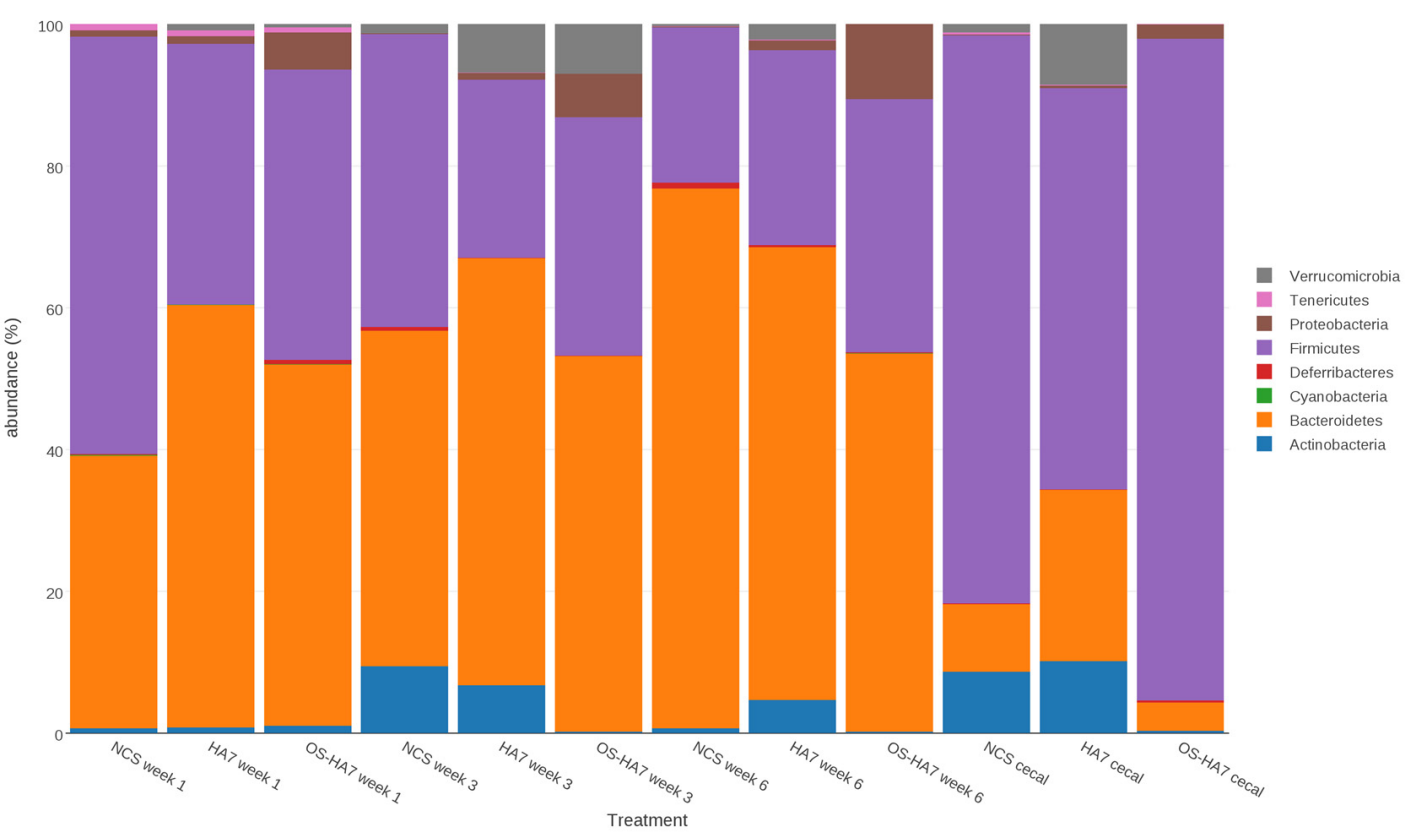
Taxonomic distribution of the bacterial phyla. The average relative distribution of the most abundant phyla is shown for mice fed control and RS diets are shown by each vertical bar. Bacterial phyla are as indicated on the right.

DNA sequences representing fecal samples from weeks 1, 3 and 6 were analyzed by QIIME and the results assessed to detect and compare the overall changes in relative abundance of the distinct taxonomic units found within the samples. The cecal contents were also recovered at the termination of the study and analyzed. As summarized in Fig 2, multiple phyla showed distinct changes in abundance in the samples when compared among all of the diet groups over the course of the six-week study. To better understand the changes, we analyzed the data through multiple comparisons.

**Fig 3:**
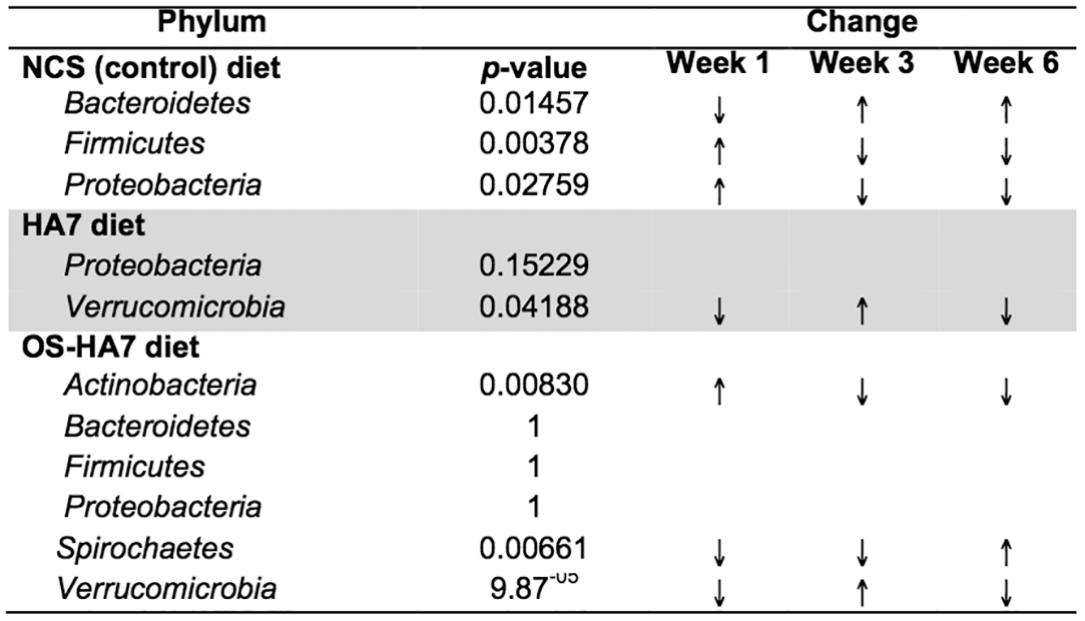
*p*-values for phyla with significant changes (Kruskal-Wallis) in relative abundance within the feces for each diet over time. Phyla with *p*-values >0.05 are given for comparison. Arrows indicate if members of the phyla are more (↑) or less (↓) abundant over the time course.

We first observed that over the course of the 6- week study, the taxonomic shifts were dynamic and distinct phyla varied in abundance over time. Phyla with statistically significant changes in abundance (Kruskal-Wallis test) from weeks 1 through 6 were identified (Fig 3). In general, the control NCS diet induced more temporal changes in the microbiota than the other two diets. Mice fed the NCS diet corresponded with an increase in *Bacteroidetes* (*p* = 0.01457) and decreases in *Firmicutes* and *Proteobacteria* (*p* = 0.0378 and 0.02759, respectively) over the 6 week period. Interestingly, during the same time the *Bacteroidetes* and *Firmicutes* were stable in the RS fed mice as the ratio of their relative abundances did not change significantly. While both RS diets altered the abundance of the *Verrucomicrobia*, the changes did not follow a single trend throughout the study, providing evidence that the relative abundance of taxa could be restored after initial diet-induced shifts. Specifically, members of the phyla were elevated at week 3 in mice fed HA7 and OS-HA7, which were restored to levels more similar to week 1 by week 6. OS-HA7 also changed the abundances of *Actinobacteria* (decrease after week 1, *p* = 0.00830) and *Spirochaetes* (increase after week 1, *p* = 0.00661).

Changes at the phylum level were observed in the cecal contents, but were limited to the OS-HA7 fed group (data not shown). Compared to the NCS diet, there were no significant changes in phyla in the HA7-fed group, while the OSHA7-fed group saw a decrease in *Actinobacteria* (*p* = 0.0056) and an increase in *Firmicutes* (*p* = 0.0056).

We also looked at the class and genus levels to identify members of the microbiota whose abundances changed over the 6 weeks for each diet (data not shown). In the NCS fed control mice, *Bacteroidia* increased over from weeks 1 to 6 (*p* = 0.023), while *Bacilli* decreased during the same period (*p* = 0.0031). Consistent with the phylum-level data Fig 2), the abundance of the *Actinobacteria* increased in week 3 but by week 6 had decreased to levels observed at week 1 (*p* = 0.31). In the HA7 group, *Bacilli* and *Gammaproteobacteria* decreased (*p* = 0.00098 and 0.020, respectively) from week 1 to week 6 (*p* = 0.00098), while *Betaproteobacteria* and *Alphaproteobacteria* increased over the same time course (*p* = 0.0087 and 0.034, respectively).

The OSHA7-fed group remained relatively stable over the six-weeks, with no significant changes in *Bacteriodia* or *Gammaproteobacteria* (*p* = 1.000 for both taxa). *Bacilli* saw a similar decrease by week 6 that was seen in the other two groups (*p* = 0.0015). *Verrucomicrobia* followed a similar trend of increasing by week 3, followed by a decrease by week 6 (*p* = 0.00015).

At the genus level, the NCS control diet animals showed an overall decrease in *Adlercreutzia* (*p =* 0.018), *Prevotela* (*p* = 0.024), and *Lactobacillus* (*p* = 0.0045). In animals fed HA7, *Bifidobacterium* and *Sutterella* increased in HA7 (*p* = 0.091 and *p* = 0.021, respectively) and *Lactobacillus* decreased after week 1 (*p* = 0.0049, data not shown).

The two dominant members in the phylum *Bacteroidetes* in the OS-HA7 group were *Bacteroides* and an unknown genus in the candidate family S24-7. In week 1, S24-7 was in greater abundance than *Bacteroides*, but after the first week this ratio was inverted so that *Bacteroides* was in greater abundance (*p* = 0.01 and 0.0076, respectively). As observed with the other two diets, *Lactobacillus* decreased from week 1 to week 6 (*p* = 0.011).

### Relative abundance of bacterial taxa change with diet

**Fig 4:**
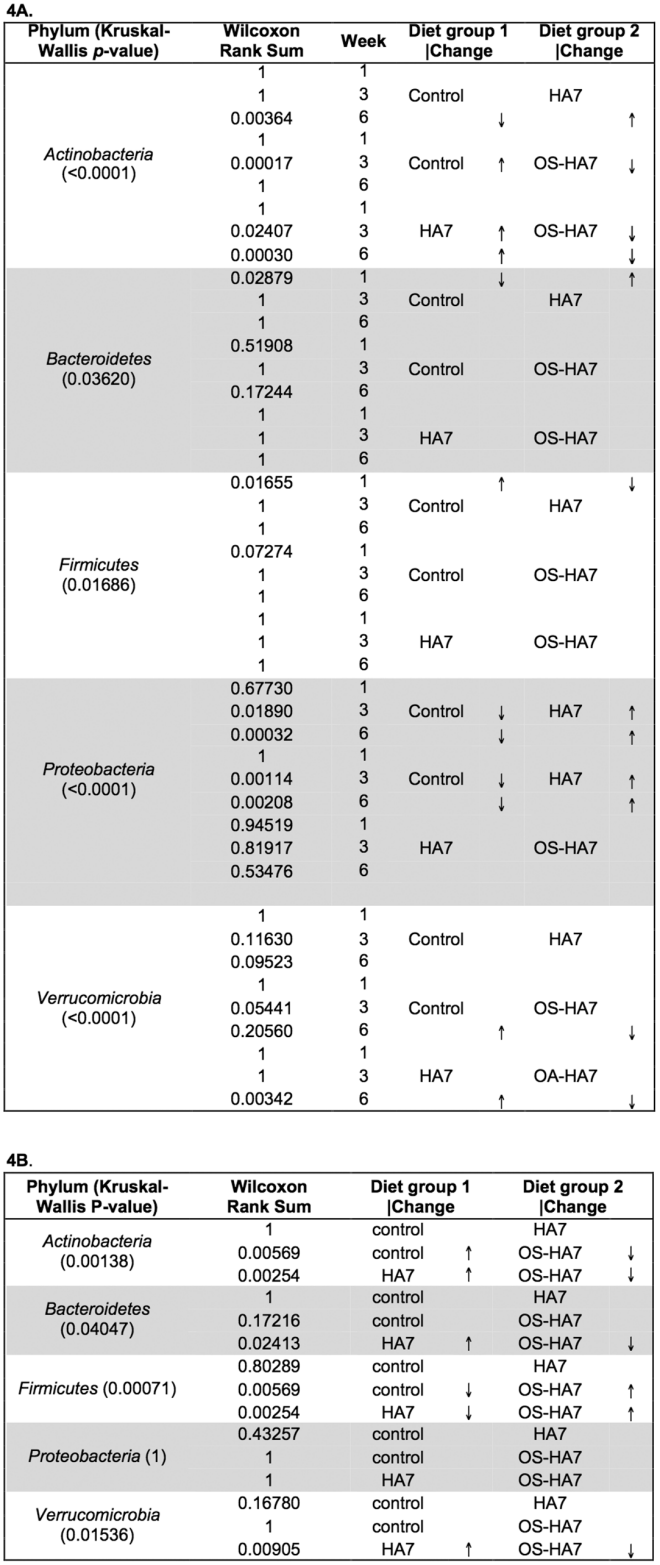
*p*-values for the differences (Kruskal-Wallis and Wilcoxon Rank Sum tests) in relative abundance of phyla. Samples from feces (4A) and cecal contents (4B) were analyzed from weeks 1, 3, and 6 for each diet. Arrows indicate if members of the phyla are more (↑) or less (↓) abundant in pairwise comparisons.

We next identified taxa that showed significant changes when abundance data from each diet were analyzed by pairwise comparisons. The *p*-values (Kruskal-Wallis test) for these comparison are shown in Fig 4A (feces) and 4B (cecum) and reveal that the *Actinobacteria*, *Bacteroidetes*, *Firmicutes*, *Proteobacteria*, and *Verrucomicrobia* showed statistically significant (*p*< 0.05) changes in abundance in the feces between the treatment groups. Similar changes were observed in the cecum as the *Actinobacteria*, *Bacteroidetes*, *Firmicutes*, and *Verrucomicrobia* differed significantly.

To more specifically identify how the diets affect the changes in taxa, pairwise comparisons between each of the diet groups were performed at each time point using Wilcoxon rank sum tests. As shown in Fig 2, each of the treatment groups showed a distinct restructuring of the gut bacterial taxa that was unique to each diet. Notably, we observed that the abundances of *Bacteroidetes* and *Firmicutes*, representing the most prevalent phyla found in all diet groups, differed more substantially between mice fed control (NCS) and either HA7 or OS-HA7 than between mice fed the RS diets. Also, changes to the phyla occurred in week 1 indicating a near immediate impact of the diets on these major taxonomic groups.

Differences were observed for the *Bacteroidetes* (*p* = 0.02879) and the *Firmicutes* (*p* = 0.01655) between the NCS and HA7 diets (Fig 4A). Members of these two phyla were redistributed so that the *Bacteroidetes* became significantly higher in the HA7 fed group, while the *Firmicutes* was lower in this RS group when compared to the NCS diet. These differences were not seen when the control group was compared with the OS-HA7 diet group, indicating HA7 and OS-HA7 did not impact the microbiota in the same way for the most abundant taxa.

Each diet also uniquely impacted other phyla, but these changes took longer to manifest. Members of the *Actinobacteria*, for example, increased in abundance beginning at week 3 in the HA7 diet group compared to other groups (e.g., *p* = 0.00364 compared to NCS diet at week 6), while OS-HA7 promoted a decrease in the same phylum when compared to the other two groups beginning at week 3. The OS-HA7 diet also presided over a decrease in *Verrucomicrobia* by week 6 (*p* = 0.00342 vs. HA7). Both RS diets promoted an increase in the *Proteobacteria* compared to the control diet group after week 1, but the changes in abundance were not significantly different between the two RS diets.

Significant changes in the abundance of selected phyla were also observed in the cecal contents throughout the study and these changes were at times different from that observed in the feces. For example, while the major phyla within the cecum included the *Firmicutes* and *Bacteroidetes*, the *Firmicutes* dominated in the cecum when compared to the feces (Figs 2 and 3). In general, the OS-HA7 diet promoted the most significant changes in abundance of selected phyla in the cecum. For example, after 6 weeks the OS-HA7 diet resulted in an increase in the *Firmicutes* compared to the control (*p* = 0.0569) and HA7 (*p* = 0.00254) diets (Fig 4B). In contrast, the same RS diet was associated with a decrease in *Actinobacteria*, *Bacteroidetes*, and *Verrucomicrobia* when compared to the other two treatment groups. Unlike the fecal samples (Fig 4A), no significant differences in the *Proteobacteria* were observed in the cecum (Fig 4B).

**Fig 5:**
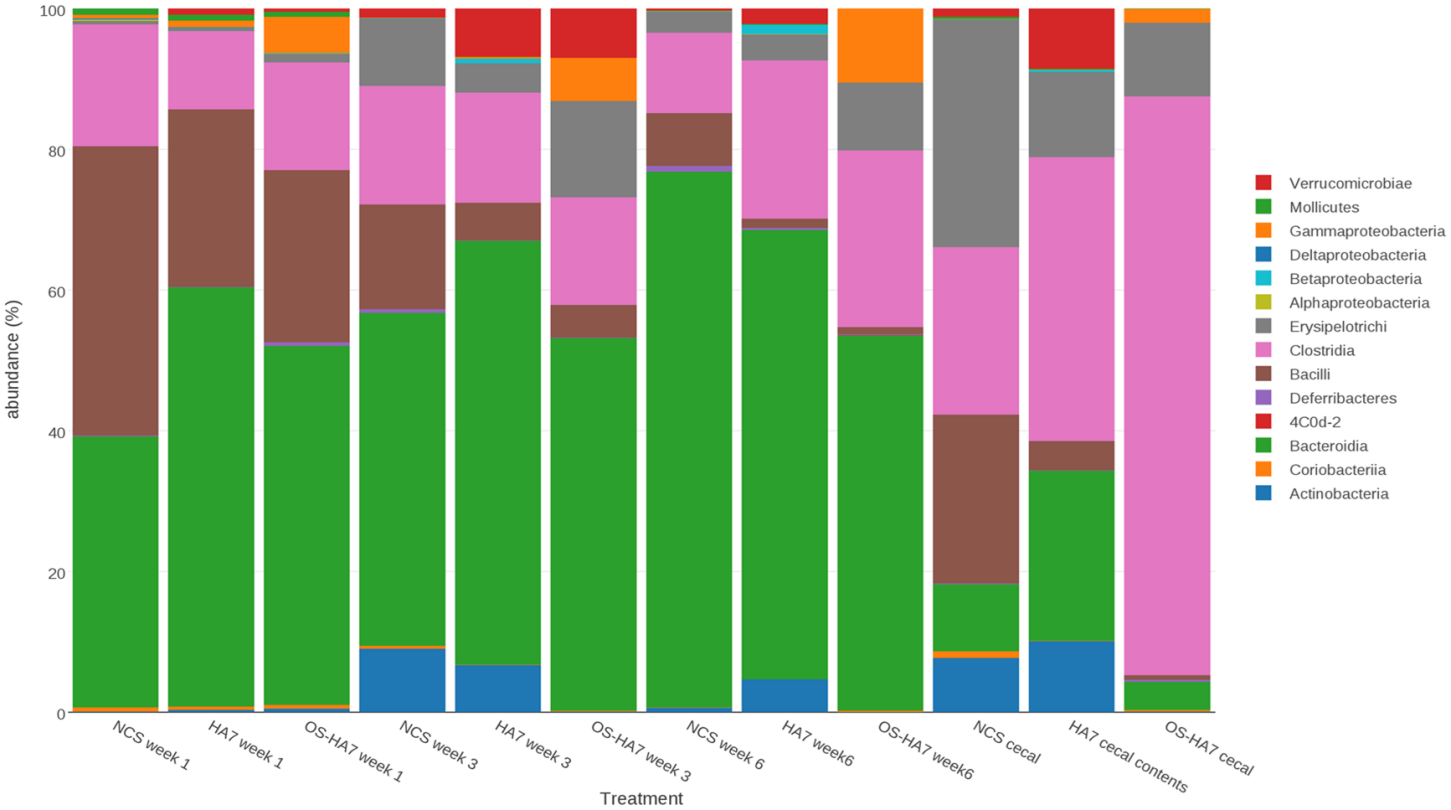
Taxonomic distribution of the bacterial classes. The average relative distribution of the most abundant classes is shown for mice fed control and RS diets are shown by each vertical bar. Bacterial classes are as indicated on the right.

**Fig 6:**
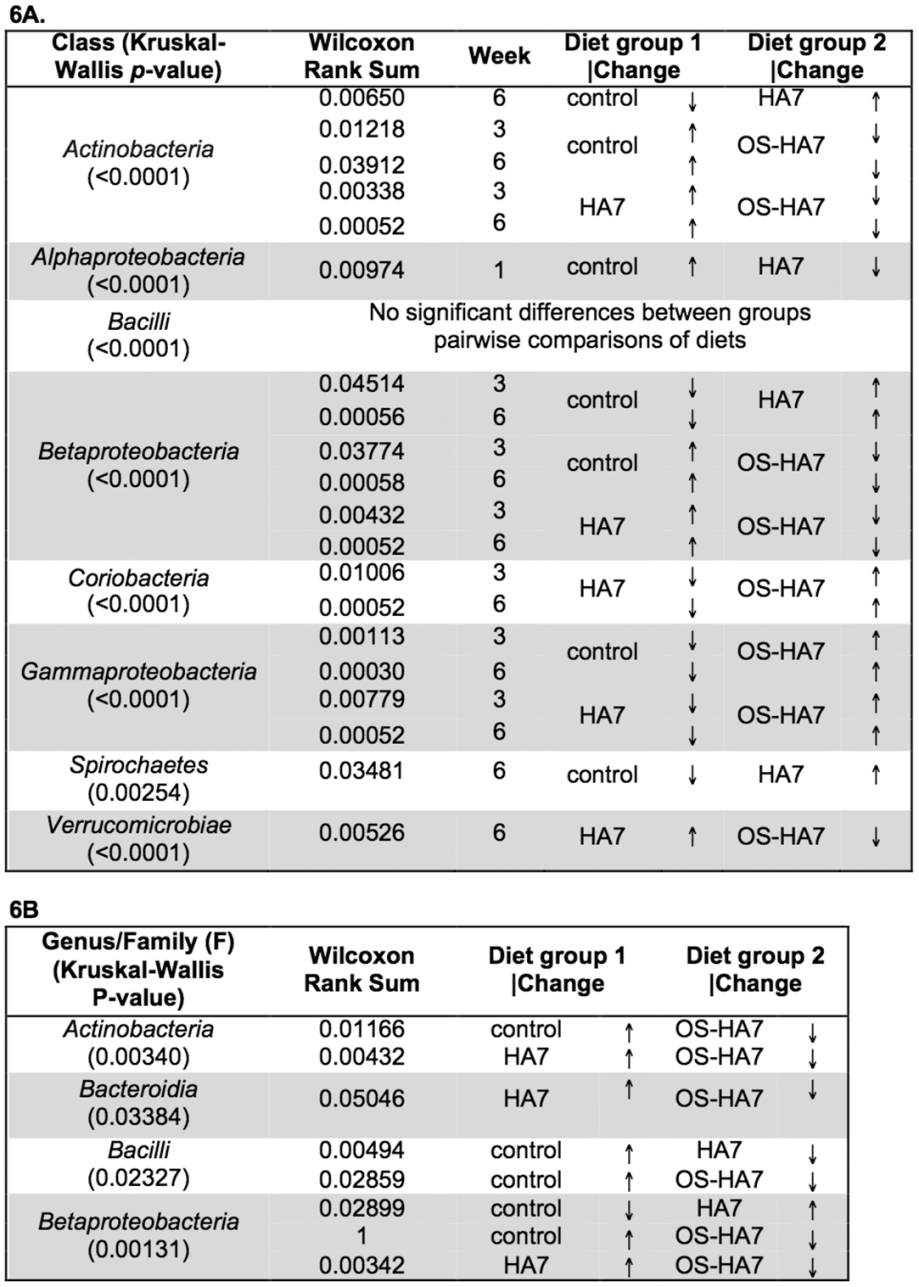
*p*-values for the differences (Kruskal-Wallis and Wilcoxon Rank Sum tests) in relative abundance of class. Samples from feces (6A) and cecal contents (6B) were analyzed from weeks 1, 3, and 6 for each diet. Only those taxa with significant *p*-values are shown. Arrows indicate if members of the phyla are more (↑) or less (↓) abundant in pairwise comparisons.

To more specifically identify the taxa with altered abundance, we identified changes at the class level associated with the different diets. Fig 5 shows the taxa summaries at the class level with Fig 6 showing the *p*-values for the overall changes in abundance in feces (6A) and cecum (6B) that were considered significant by the Kruskal-Wallis test as well as pairwise comparisons by Wilcoxon Rank Sum tests. Comparing the different diets by weeks at the class level revealed that, with the exception of *Alphaproteobacteria*, no significant differences between the NCS control starch and the RS diets occurred in week 1. Consistent with the phylum-level results, members of the *Actinobacteria*, *Alpha-*, *Beta-* and *Gamma-proteobacteria*, and *Verrucomicrobia* showed significant differences in diet comparisons after week 1 in the feces (Fig 6A). By week 6, *Actinobacteria* differed in all pairwise diet comparisons (control:HA7 *p* = 0.0065, control:OSHA7 *p* = 0.03912, HA7:OSHA7 *p* = 0.00052), with HA7 having the highest and the OS-HA7 group having the lowest abundance. *Betaproteobacteria* followed the same pattern with all pairwise comparisons significantly different from each other and HA7 having the greatest abundance and OS-HA7 having the lowest (control:HA7 *p* = 0.00056, control:OSHA7 *p* = 0.00058, HA7:OSHA7 *p* = 0.00052). Also, *Gammaproteobacteria* was enriched in the OSHA7 group when compared to both the control and HA7 groups (control:OSHA7 *p* = 0.0030, HA7:OSHA7 *p* = 0.00052).

**Fig 7:**
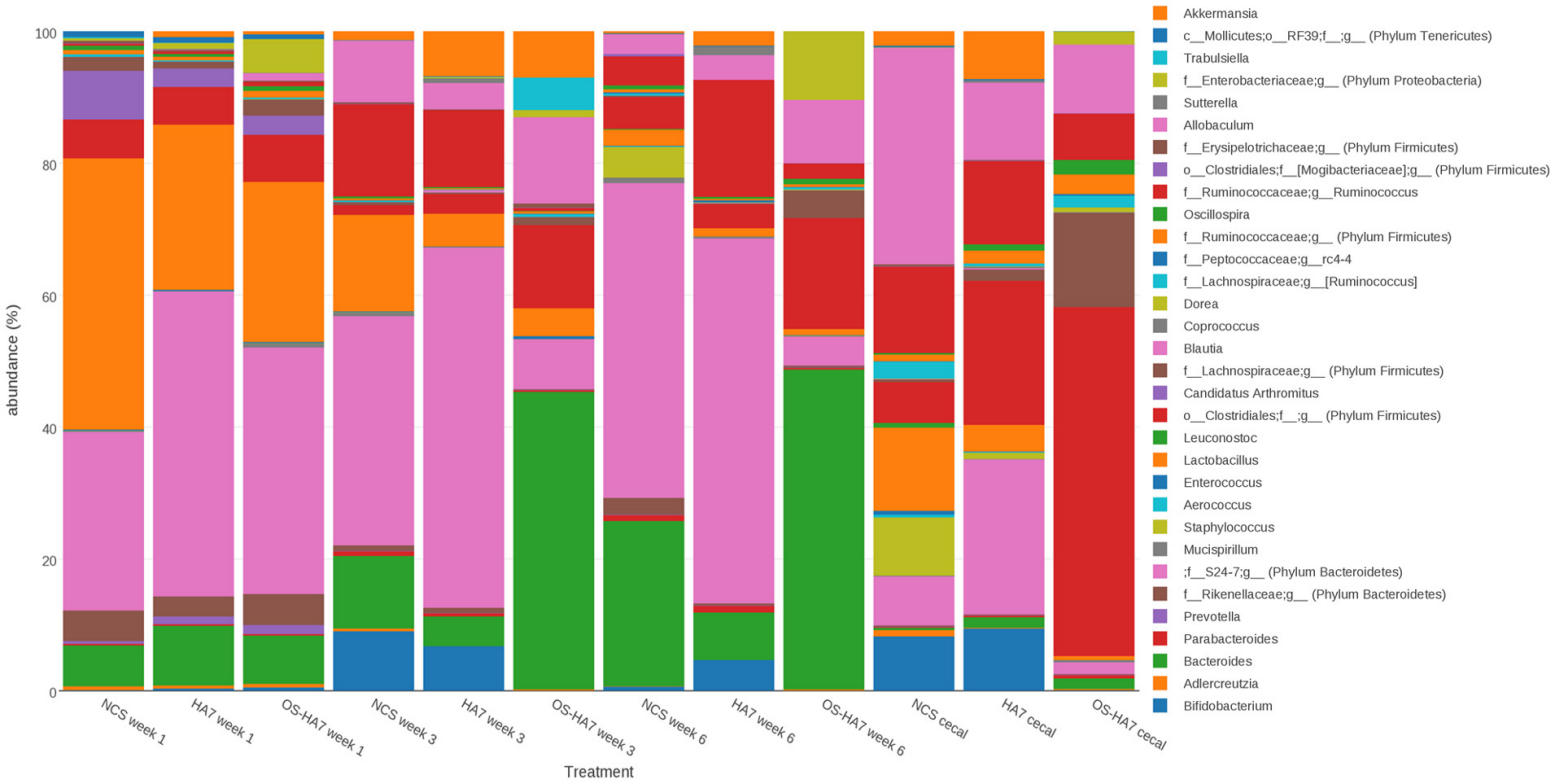
Taxonomic distribution of the bacterial genera. The average relative distribution of the most abundant genera is shown for mice fed control and RS diets are shown by each vertical bar. Bacterial genera are as indicated on the right.

**Fig 8:**
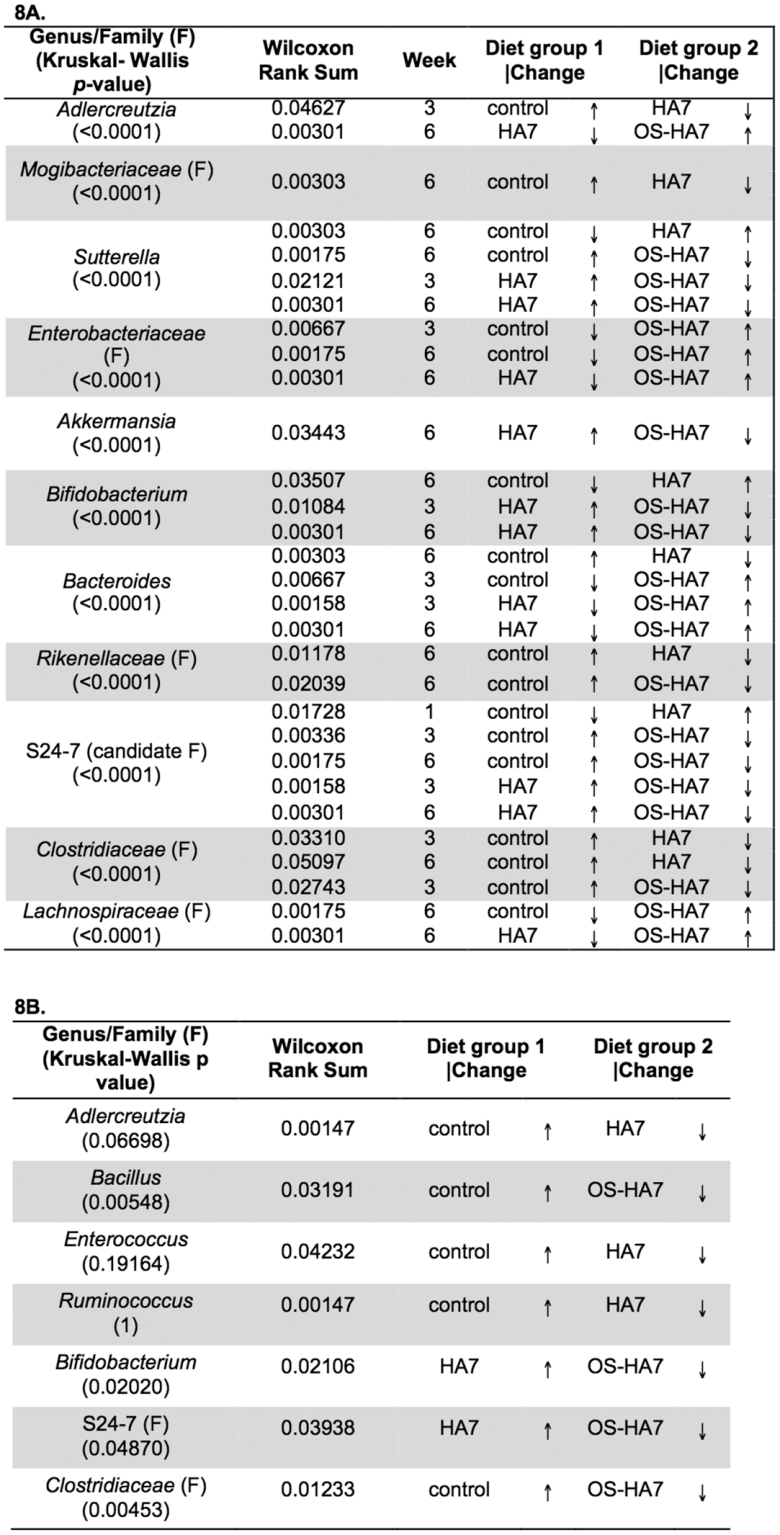
*p*-values for the differences (Kruskal-Wallis and Wilcoxon Rank Sum tests) in relative abundance of genus (or family). Samples from feces (8A) and cecal contents (8B) were analyzed from weeks 1, 3, and 6 for each diet. Only those taxa with significant *p*-values are shown. Arrows indicate if members of the phyla are more (↑) or less (↓) abundant.

Several genera whose abundances were altered by the diets after week 1 were also identified (Figs 7 and 8). Comparisons of the diet in week 1 revealed few differences among the diets at the genus level. By week 3, however, an increased number of genera showed differences among the groups. For example, compared to the control diet, *Aldercreutzia* and *Bacteroides* were depleted in the HA7 group (*p* = 0.04627 and *p* = 0.00303, respectively), while *Sutterella* (Phylum *Proteobacteria*), and *Bifidobacterium* showed significant increases (*p* = 0.00303 and 0.03507, respectively). Inspection of Fig 8A shows that in the pairwise diet comparisons, most of the changes of note were associated with the OS-HA7 diet, both when compared to the control diet, as well as HA7. Comparison of the cecal contents revealed several genera consistently showed a decrease in abundance with the RS diets (Fig 8B).

### Alpha and Beta Diversity

For the Shannon (alpha) diversity metrics we used a nonparametric two-sample t-test using Monte Carlo permutations to determine the extent to which groups differed from each other. Comparing Shannon diversity values (S1 Table) for each group and determining statistical significance (S2A Table) revealed that none of the diet-groups differed significantly from each other by week. No significant differences in alpha diversity at week 1 were observed within each diet (*p* = 0.066). Likewise, the diversity among the different diets within weeks 3 and 6 did not differ from each other. Inspection of number of OTUs discovered versus number of sequence reads revealed that the sequencing depth was sufficient to exclude discovery of new or rare OTUs (S1A Fig). Nonparametric two-sample t-tests revealed there was no significant change in OTU discovery per sequence reads among the groups at each week (S2B Table). Statistical analysis (S2C Table) of Faith’s Phylogenetic Diversity (S1B Fig) revealed that the overall microbial diversity was not significantly altered by the diets.

**Fig 9:**
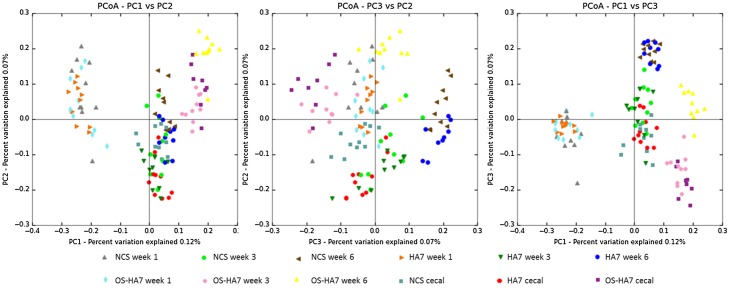
Global effects of diet on mouse gut microbiota. Unweighted UniFrac distance PCoA plots comparing three principal components are shown. Samples representing each diet and time point of collection are indicated by distinct symbols.

QIIME was also used to generate PCoA plots of the unweighted distances obtained from the beta diversity workflow. Fig 9 shows the unweighted UniFrac PCoA plots and the clustering of the groups, where each point represents an individual sample. Analysis of similarity (ANOSIM) and Adonis tests were performed on the unweighted distances to determine the similarity and dissimilarity of the groups. The ANOSIM *p*-value of 0.001 and an R-value of 0.836, which is close to 1.0, indicate dissimilarity among the groups. A *p*-value of 0.001 in the Adonis test also revealed that the clustering of the groups is significant and the groups were dissimilar in composition. An R^2^ value of 0.37712 signified that 37.712% of the variation in the groups can be explained by this clustering.

### Mice Treated with HA7 Demonstrated Increased Anxiety-like Behavior on the Elevated Plus Maze

**Fig 10:**
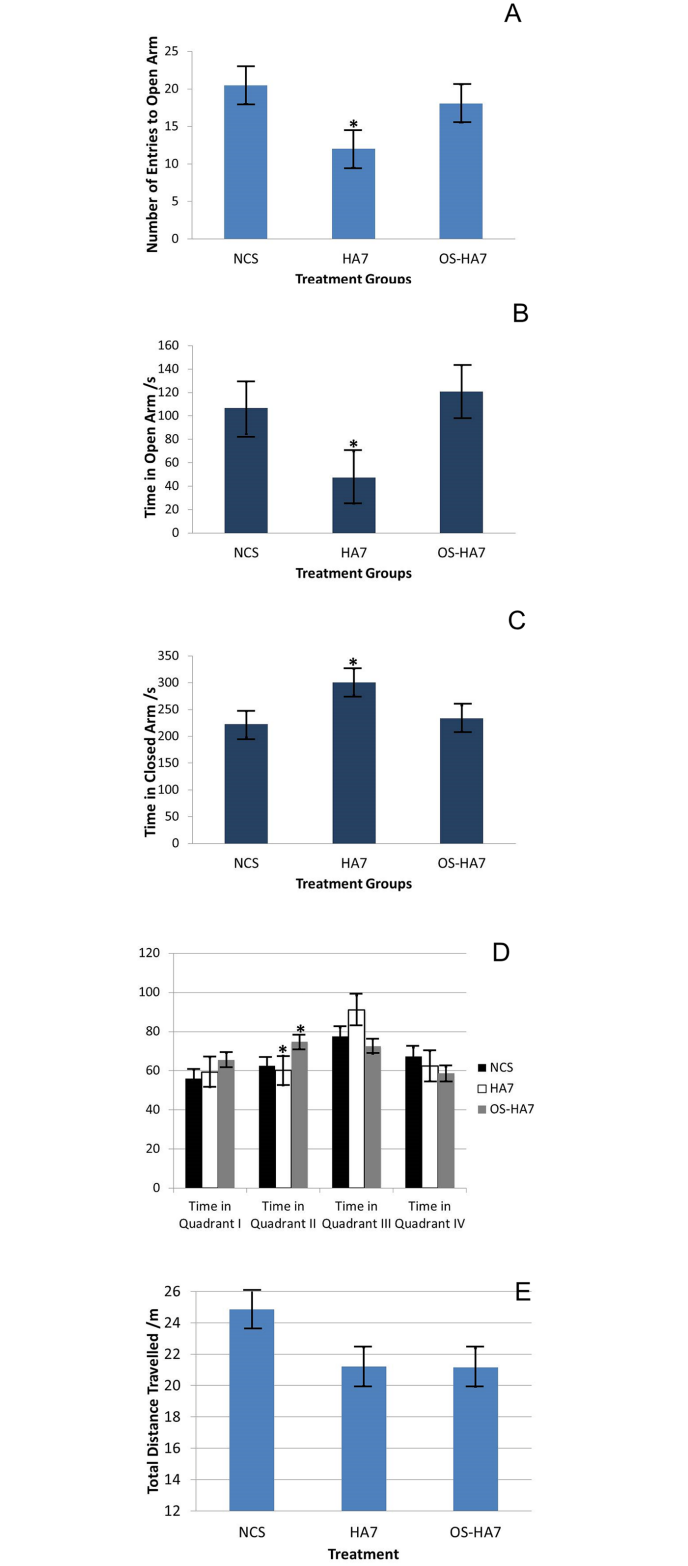
Behavioral and locomotion tests. 10A. The number of entries to the open arm for the three groups. There were no significant differences detected between NCS and OS-HA7 or HA7 and OS-HA7, but there were significant differences between NCS and HA7. Values represent the mean number of entries to open arm in each group ± SEM, n = 16/group. *p<0.05. 10B. Time spent in the open arm for the three groups. There were no significant differences detected between NCS and OS-HA7, but there were significant differences between NCS and HA7 and OS-HA7 and HA7. Values represent the mean time spent in the open arm in each group ± SEM, n = 16/group. *p<0.05. 10C. Time spent in the closed arm for the three groups. There were no significant differences detected between NCS and OS-HA7, but there were significant differences between NCS and HA7 and OS-HA7 and HA7. Values represent the mean time spent in the closed arm in each group ± SEM, n = 16/group. *p<0.05. 10D. Overall locomotion of mice during the open field test (10D). Values represent the mean time spent in each quadrant in each group ± SEM, n = 16/group. *p<0.05. 10E. Overall locomotion of mice during the open field test (10E). Values represent the mean distance travelled ± SEM, n = 16/group.

Investigation of changes in anxiety-like behavior were observed through the EPM. In mice treated with the HA7 diet, there was a significant reduction in the number of entries into the open arm (Fig 10A; NCS:HA7 *p* = 0.041; HA7:OS-HA7 *p* = 0.221) as well as the time spent in the open arm (Fig 10B; NCS:HA7 *p* = 0.004; HA7:OS-HA7 *p* <0.0001) with a subsequent increase in the time spent in the closed arm (Fig 10C; NCS:HA7 *p* = 0.006; HA7:OS-HA7 *p* = 0.019). In contrast, mice treated with NCS and OS-HA7 spent more time in the open arm and less time in the closed arm. No differences were observed between the mice treated with NCS and those treated with OS-HA7 in the number of entries into the open arm (NCS:OS-HA7 *p* = 1.000), time spent in the open arm (NCS:OS-HA7 *p* = 1.000) and time spent in the closed arm (NCS:OS-HA7 *p* = 1.000). HA7 fed mice spent a signficantly greater time grooming (*p*< 0.05) compared to NCS fed mice.

### Mice Treated with HA7 and OS-HA7 Demonstrated Decreased Exploration on the Open Field

In addition to the elevated plus maze, the open field was used to test for differences in exploration. Overall, there were no significant differences in locomotor behavior in the open field apparatus, however there were statistically significant differences in exploration as demonstrated by transitions between quadrants. Mice treated with NCS transitioned between quadrants more than mice treated with HA7 or OS-HA7 (NCS:HA7 p = 0.023; NCS:OS-HA7 p = 0.025) with no significant differences between mice treated with HA7 and OS-HA7 (HA7:OS-HA7 p = 1.000). Furthermore, mice treated with NCS also demonstrated increased rearing compared to mice treated with HA7 and OS-HA7 (NCS: HA7 p = 0.002; NCS:OS-HA7 p<0.05) with no statistically significant differences between mice treated with HA7 and OS-HA7 (HA7:OS-HA7 p = 1.000). As shown in Fig 10D, there were no differences in the average amount of time each diet-group spent in quadrant I, III and IV during the test (QI ANOVA *p* = 0.195; QIII ANOVA *p* = 0.042; QIV ANOVA *p* = 0.406). There were statistically significant differences in the time spent in quadrant II (Fig 10D; QII ANOVA *p* = 0.006; NCS:OS-HA7 *p* = 0.033; HA7:OS-HA7 *p* = 0.008). However, these results did not appear to be biologically significant because they did not represent differences in the overall locomotor behavior as a total measure of locomotion as quantified by total distance travelled and revealed no statistically significant differences between groups (Fig 10E; ANOVA *p* = 0.055).

### Hormone Analysis of Hair Samples Revealed Differences in Corticosterone Levels

Hair samples revealed a significant difference in corticosterone levels, while all hormones showed no differences (Table 2). Analysis revealed statistically significant (NCS:HA7 *p* = 0.693; NCS:OS-HA7 *p* = 0.145; HA7:OS-HA7 *p* = 0.012) higher levels of corticosterone detected in NCS (0.028 ng/mg) and OS-HA7 (0.047 ng/mg) compared to HA7 (0.017 ng/mg).

**Table 2:** Hormone profiles in murine hair according to diet group.

Hormone	NCS		HA7		OS-HA7		ANOVA
	Mean	SD	Mean	SD	Mean	SD	
Estrone	0.0127	0.0146	0.0172	0.0125	0.0380	0.0415	0.2427
Estradiol	0.0023	0.0050	0.0014	0.0016	0.0005	0.0013	0.6117
Testosterone	0.0044	0.0012	0.0049	0.0013	0.0040	0.0018	0.5612
Progesterone	0.0046	0.0047	0.0079	0.0079	0.0091	0.0095	0.5862
Corticosterone	0.0283	0.0157	0.0173	0.0089	0.0473	0.0194	0.0128*
DHEA	0.0691	0.0405	0.0602	0.0181	0.1119	0.0713	0.1768
Androstenedione	0.0041	0.0010	0.0053	0.0014	0.0035	0.0012	0.0613

Hair samples were collected from 6 mice per group at the end of the experiment and analyzed for hormone levels. Values represent mean values for each diet and *p*-values from ANOVA tests, n = 6/group.

*p<0.05.

## Discussion

The results presented demonstrate that an RS-supplemented diet can both alter the gut microbiota composition as well as influence brain function as reflected in an increase in anxiety-related behaviors. That the interplay between diet, microbiome and behavior is a complex one is amply illustrated by the study of Ohland *et al*. [38] who observed that the ability of a probiotic bacterium to influence behavior in mice was dependent not only on the animal’s genotype, but also on the diet that was being consumed. This is consistent with our understanding that the diet supplies many of the chemical precursors that are required as part of biosynthetic pathways that are required for synthesis of neurochemicals both by the host as well as the microbiota that may influence brain function [39].

Studies continue to demonstrate in humans that the composition of the microbiota has a dramatic influence on the behavior of the individual. For example, the composition of the mucosal microbiota of cirrhotic patients was linked to poor cognition [40]. Individuals suffering Inflammatory Bowel Disease (IBD) have been recognized for decades to suffer from anxiety and depression [41]. The realization that the microbiome in IBD was altered as compared to age matched controls led to attempts to instill in “good” bacteria such as probiotics to alleviate the behavioral deficits. Messaoudi *et al*. [40] demonstrated that in physically healthy individuals, measurements of psychological distress such as self-blame, depression, or anger/hostility, could be meaningfully addressed via the administration of probiotics. Likewise, in rats, probiotics have been used to attenuate anxiety-like behaviors [42, 43]. Little is known concerning the mechanisms by which gut microbiota, either commensal organisms or administered bacteria such as probiotics, can exert psychotropic effects in the host. In general, studies have shown that the intestinal bacteria most directly affect the brain and subsequent behavior via pathways mediated by the vagus nerve [7, 44].

Starch is a major energy source in human and animal diets. *In vitro* analysis of RS contents of the three starch varieties show that after boiling NCS displays 0.8% RS content, whereas HA7 and OS-HA7 display 24.1% and 20.9% RS, respectively [27]. After the starches are mixed with casein, corn oil, and other ingredients to make rodent diets (Table 1), the RS content of the OS-HA7 diet increases about 36% compared with the starch analyzed alone while other diets remain similar. Previous studies have shown that after feeding rodents diets made from these three varieties of cooked starch demonstrated that while only 0.1% NCS was not utilized, 1.7% HA7 and 20.2% OS-HA7 were not utilized after feeding for nine weeks [27].

What are the mechanism(s) and pathway(s) by which RS-induced alterations in the microbiome influence the brain? Each diet induced specific changes in the gut microbiota. The simplest explanation is that specific microorganisms that produce metabolites, including neurochemicals, that affect brain function are altered in response to RS. The change in concentration of these metabolites consequently has a direct effect on brain activity. Our results suggest more complicated mechanisms are in play. Specifically, we observed an increase in *Bifidobacterium* species in HA7-fed mice (S1 Table). This genus has been associated with relief of anxiety in rat models of stress and mood disorders [45, 46]. Although the HA7 diet increased the abundance of *Bifidobacterium*, this increase was not able to overcome the increased anxiety induced by the same diet (Fig 10). The results here are also consistent with the study by Tachon *et al*., who showed a similar increase in *Bifidobacterium* in mice fed a type 2 RS [22]. A reduction in *Bacteroides* has been associated with mice exposed to stress from social disruption [47]. HA7 fed mice had a reduced relative abundance of *Bacteroides* compared to NCS and OS-HA7. *Bacteroides* increased in the NCS and OSHA7 diets, but remained low throughout the study in the HA7 diet. Mice fed OS-HA7 showed a distinct shift in the microbiota towards increased *Proteobacteria* (Figs 2 and 3). It is known that dysbiosis and epithelial damage and inflammation may provide a unique niche that selects for growth of *Proteobacteria* [48–50]. The unique structure of OS-HA7 may indirectly contribute to growth of *Proteobacteria* through alteration of the normal gut microbiota, or by inducing inflammation in the GI tract. The perturbation of the gut by OS-HA7 is consistent with the weight loss observed in mice fed this formulation of RS (Fig 1). It is clear that by altering of the amylose content of starch or introducing hydrophobic and bulky OS groups into starch it is possible to vary the degree of RS and hence the effect on the composition of the microbiome. Levels of hormones measured in hair samples did not significantly differ between NCS vs HA7 and NCS vs OS-HA7 for any of the analytes measured, indicating behavioral differences were not due to differences in these related hormones (Table 2).

In addition to changes in abundance among specific bacterial taxa at the end of the study, we observed that the gut microbiota were dynamically altered throughout the feeding regimens. For example *Actinobacteria* increased in week 3 before decreasing in week 6 in the NCS and HA7 groups. *Verrucomicrobia* increased in week 3 then decreased again in all groups. *Bacilli* decreased in all groups throughout the study while *Clostridia* increased in the HA7 and OS-HA7 groups.

In addition to changes in the gut microbiota, we along with others, observed that RS also alters the physiological function in the rodent cecum, as evidenced by enlarged ceca (data not shown), which is indicative of enhanced fermentation activity [22, 51, 52]. While enlargement of the cecum in rodents fed RS has been observed previously [22, 51, 52] behavioral analysis was not done in these studies. Interestingly, it has been reported that aging rats fed RS diets showed changes in behavioral activity as measured by increased eating responses to fasting and improved motor coordination [53]. It is not clear if these rodents also experienced anxiety-like behavior or if the behavioral changes were specific to the rat model or the type of RS used. While additional studies will be needed to identify the precise mechanism(s) by which the RS diets alter behavior, we predict it will be a combination of changes to multiple bacterial taxa along with physiological changes to the GI tract.

## Conclusion

Modulation of the microbiome represents an emerging therapeutic modality by which behavior may be addressed to improve quality of life [11]. The use of a targeted dietary approach through use of RS represents a means to selectively “dial-in” a specific microbiome to achieve a desired neurological outcome. The relative low-cost and high compliance of dietary interventions is an appealing alternative to the use of pharmaceutical agents [54]. While the current study indeed reinforces that RS is an effective means to achieve changes in the taxonomic profile of the gut microbiota, it also reveals the potential of RS to affect behavior [53]. The results presented here underscore the need to consider the potential that certain types of RS have for inducing, rather than ameliorating, anxiety-like behaviors when designing experiments to test the potential for digestion-resistant starches to improve health outcomes.

## Supporting Information

S1 Fig Rarefaction plots of observed species richness alpha diversity (S1A) and Faith’s Phylogenetic Diversity (S1B). (DOCX)

S1 Table Shannon diversity values. Values are given for each sample where samples with an “R” represents mice fed the control starch, “B” mice fed HA7, and “G” mice fed OS-HA7.
(XLSX)

S2 Table Statistical significance of Shannon diversity (S2A), species richness (S2B) and Faith’s Phylogenetic Diversity values (S2C). (PDF)
