# Supplementary material for: Resistant Starch Alters the Microbiota-Gut Brain Axis: Implications for Dietary Modulation of Behavior
Source: PLoS One. 2016 Jan 8;11(1):e0146406. doi: 10.1371/journal.pone.0146406 (PMC4706316; doi:10.1371/journal.pone.0146406)

**Figure S1. Rarefaction plots of observed species richness alpha diversity (S1A) and Faith’s Phylogenetic Diversity (S1B).** Each line represents a single diet, as shown by line color. The plateau of the curves indicates the depth of the sequencing was sufficient enough to preclude discovery of new or rare OTUs.

**S1A**


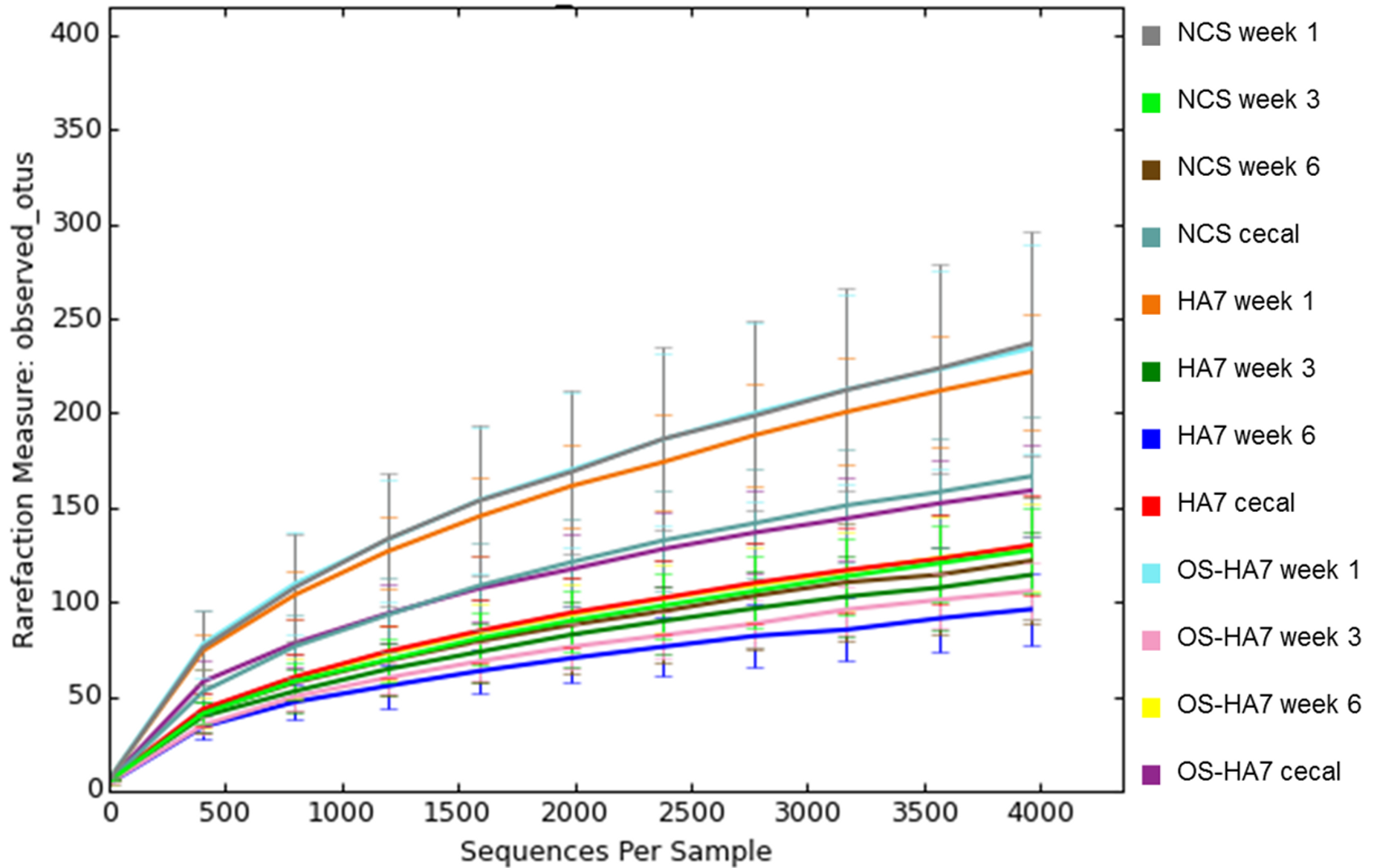


**S1B**


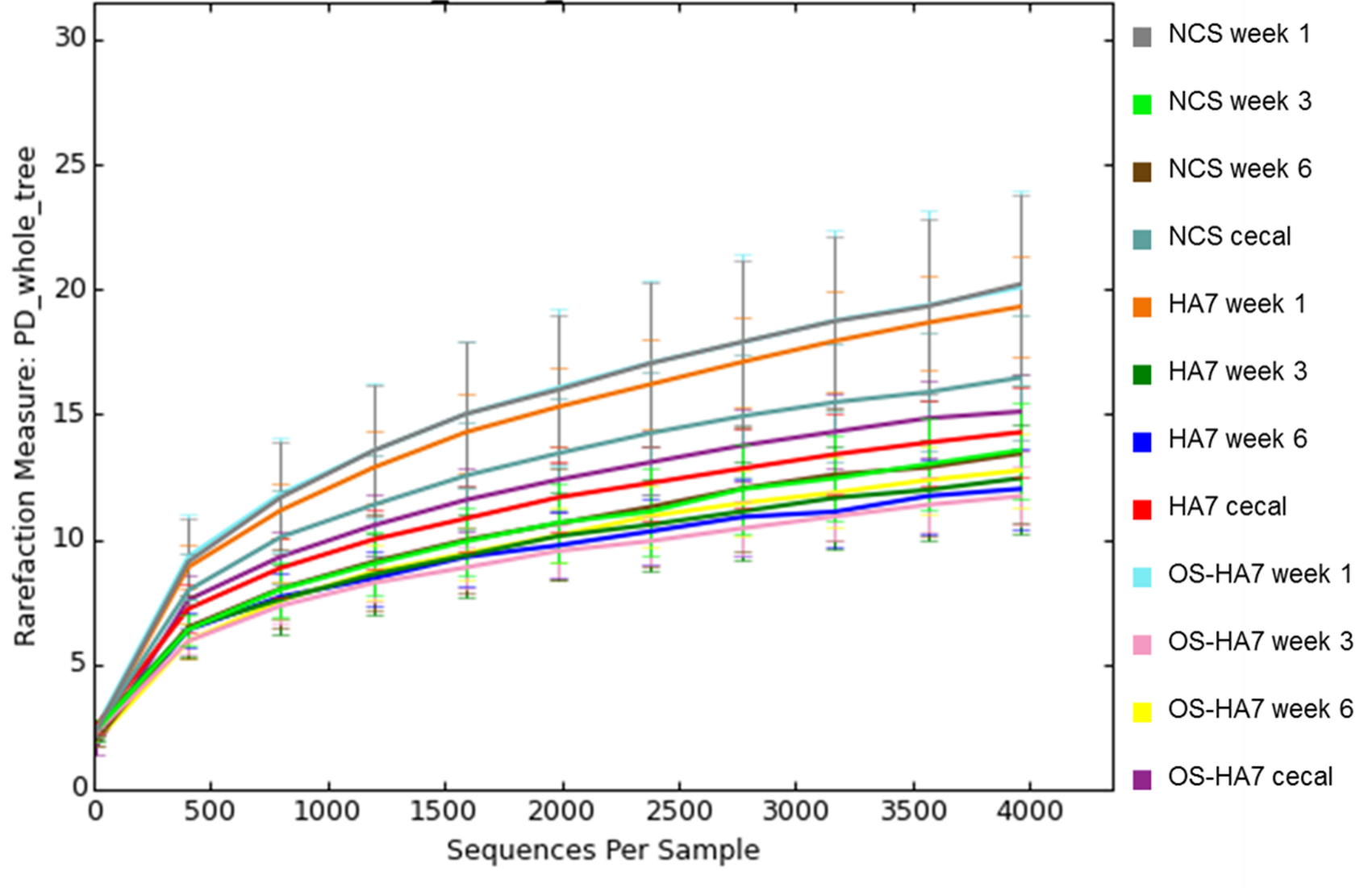

Supplement: S1 Fig — (DOCX) [file pone.0146406.s001.docx]
