# Supplementary material for: Resistant Starch Alters the Microbiota-Gut Brain Axis: Implications for Dietary Modulation of Behavior
Source: PLoS One. 2016 Jan 8;11(1):e0146406. doi: 10.1371/journal.pone.0146406 (PMC4706316; doi:10.1371/journal.pone.0146406)
Supplement: S2 Table — (PDF) [file pone.0146406.s003.pdf]

Table S2A: Statistical significance of Shannon diversity values

Group 1 Diet/week	Group 2 Diet/week	Group1 mean	Group1 std*	Group2 mean	Group2 std*	t stat**	*p*-value
NCS/3	NCS/1	3.77786	0.22886	4.88775	0.5192	-5.86799	0.066
NCS/6	NCS/1	3.724333	0.37251	4.88775	0.5192	-5.46177	0.066
NCS/6	NCS/3	3.724333	0.37251	3.77786	0.2288	-0.36730	1
HA7/1	HA7/3	5.229007	0.27573	3.60795	0.4977	8.546266	0.066
HA7/6	HA7/1	2.835618	0.68188	5.22900	0.2757	-9.65830	0.066
HA7/6	HA7/3	2.835618	0.68188	3.60795	0.4977	-2.68510	1
OS-HA7/1	OS-HA7/3	5.152535	0.67151	3.03713	0.4058	8.088071	0.066
OS-HA7/6	OS-HA7/1	3.167414	0.61127	5.15253	0.6715	-6.55824	0.066
OS-HA7/6	OS-HA7/3	3.167414	0.61127	3.03713	0.4058	0.532652	1
HA7/1	NCS/1	5.229007	0.27573	4.88775	0.5192	1.741416	1
OS-HA7/1	NCS/1	5.152535	0.67151	4.88775	0.5192	0.935815	1
HA7/1	OS-HA7/1	5.229007	0.27573	5.15253	0.6715	0.316034	1
HA7/3	NCS/3	3.607959	0.49776	3.77786	0.2288	-0.93036	1
OS-HA7/3	NCS/3	3.037137	0.40585	3.77786	0.2288	-4.76923	0.066
HA7/3	OS-HA7/3	3.607959	0.49776	3.03713	0.4058	2.66632	1
HA7/6	NCS/6	2.835618	0.68188	3.72433	0.3725	-3.37840	0.33
OS-HA7/6	NCS/6	3.167414	0.61127	3.72433	0.3725	-2.33397	1
HA7/6	OS-HA7/6	2.835618	0.68188	3.16741	0.6112	-1.05789	1
OS-HA7/ cecal	NCS/cecal	4.192789	0.66901	3.91405	0.5526	0.912534	1
OS-HA7/ cecal	HA7/cecal	4.192789	0.66901	3.80372	0.4735	1.359573	1
HA7/cecal	NCS/cecal	3.803721	0.47358	3.91405	0.5526	-0.45480	1

*standard deviation

** t test statistic

Table S2B. Statistical significance of observed species richness values.

Group 1 Diet/week	Group 2 Diet/week	Group1 mean	Group1 std*	Group2 mean	Group2 std*	t stat**	*p*-value
NCS/3	NCS/1	127.3	22.22561	236.7	59.17093	-5.192428	0.066
NCS/6	NCS/1	121.77	33.40748	236.7	59.17093	-5.074140	0.066
NCS/6	NCS/3	121.77	33.40748	127.3	22.22561	-0.413455	1
HA7/1	HA7/3	221.84	30.33075	114.33	22.9222	8.4835655	0.066
HA7/6	HA7/1	96.144	18.5096	221.84	30.33075	-10.17739	0.066
HA7/6	HA7/3	96.144	18.5096	114.33	22.9222	-1.787189	1
OS-HA7/1	OS-HA7/3	234.04	55.40967	105.67	14.78411	6.7153074	0.066
OS-HA7/6	OS-HA7/1	129.3	23.10908	234.04	55.40967	-5.233902	0.066
OS-HA7/6	OS-HA7/3	129.3	23.10908	105.67	14.78411	2.58406	1
HA7/1	NCS/1	221.84	30.33075	236.7	59.1709	-0.670458	1
OS-HA7/1	NCS/1	234.04	55.40967	236.7	59.17093	-0.098440	1
HA7/1	OS-HA7/1	221.84	30.33075	234.04	55.40967	-0.579407	1
HA7/3	NCS/3	114.33	22.9222	127.3	22.22561	-1.218673	1
OS-HA7/3	NCS/3	105.67	14.78411	127.3	22.22561	-2.43092	1
HA7/3	OS-HA7/3	114.33	22.9222	105.67	14.78411	0.952472	1
HA7/6	NCS/6	96.144	18.5096	121.77	33.40748	-1.926749	1
OS-HA7/6	NCS/6	129.3	23.10908	121.77	33.40748	0.5561121	1
HA7/6	OS-HA7/6	96.144	18.5096	129.3	23.10908	-3.241695	0.198

*standard deviation
** t test statistic

Table S2C. Statistical significance of Faith's Phylogenetic Diversity values.

Group 1 (Diet/week)	Group 2 (Diet/week)	Group1 mean	Group1 std*	Group2 mean	Group2 std*	t stat**	*p*-value
NCS/3	NCS/1	13.56233	1.95248	20.20653	3.57882	-4.8893	0.066
NCS/6	NCS/1	13.43341	2.73107	20.20653	3.57882	-4.5135	0.132
NCS/6	NCS/3	13.43341	2.73107	13.56233	1.95248	-0.1152	1
HA7/1	HA7/3	19.30618	2.02150	12.44779	2.17346	6.9317	0.066
HA7/6	HA7/1	12.02321	1.65788	19.30618	2.02150	-8.0690	0.066
HA7/6	HA7/3	12.02321	1.65788	12.44779	2.17346	-0.4491	1
OS-HA7/1	OS-HA7/3	20.08043	3.88240	11.73642	1.18708	6.16579	0.066
OS-HA7/6	OS-HA7/1	12.75765	1.49566	20.08043	3.88240	-5.2801	0.066
OS-HA7/6	OS-HA7/3	12.75765	1.49566	11.73642	1.18708	1.60444	1
HA7/1	NCS/1	19.30618	2.02150	20.20653	3.57882	-0.6571	1
OS-HA7/1	NCS/1	20.08043	3.88240	20.20653	3.57882	-0.0716	1
HA7/1	OS-HA7/1	19.30618	2.02150	20.08043	3.88240	-0.5306	1
HA7/3	NCS/3	12.44779	2.17346	13.56233	1.95248	-1.1444	1
OS-HA7/3	NCS/3	11.73642	1.18708	13.56233	1.95248	-2.3972	1
HA7/3	OS-HA7/3	12.44779	2.17346	11.73642	1.18708	0.86174	1
HA7/6	NCS/6	12.02321	1.65788	13.43341	2.73107	-1.2697	1
OS-HA7/6	NCS/6	12.75765	1.49566	13.43341	2.73107	-0.6510	1
HA7/6	OS-HA7/6	12.02321	1.65788	12.75765	1.49566	-0.9602	1
OS-HA7/ cecal	NCS/cecal	14.28590	1.77195	16.47021	2.48448	-2.1473	1
OS-HA7/ cecal	HA7/cecal	15.11309	1.54492	16.47021	2.48448	-1.2730	1
HA7/cecal	NCS/cecal	15.11309	1.54492	14.28590	1.77195	0.98166	1

*standard deviation
** t test statistic
